# Two Intercalation Mechanisms of Oxazole Yellow Dimer (YOYO-1) into DNA

**DOI:** 10.3390/molecules26123748

**Published:** 2021-06-19

**Authors:** Karolina Kucharska, Marta Pilz, Krzysztof Bielec, Tomasz Kalwarczyk, Patrycja Kuźma, Robert Hołyst

**Affiliations:** Department of Soft Condensed Matter, Institute of Physical Chemistry PAS, Kasprzaka 44/52, 01-224 Warsaw, Poland; karolex.kk@gmail.com (K.K.); marta.pilz15@gmail.com (M.P.); bielec.krz@gmail.com (K.B.); t.kalwarczyk@gmail.com (T.K.); patrycja.kuzma7@wp.pl (P.K.)

**Keywords:** DNA, YOYO-1, brightness method, equilibrium constant, intercalation

## Abstract

The oxazole yellow dye, YOYO-1 (a symmetric homodimer), is a commonly used molecule for staining DNA. We applied the brightness analysis to study the intercalation of YOYO-1 into the DNA. We distinguished two binding modes of the dye to dsDNA: mono-intercalation and bis-intercalation. Bis-intercalation consists of two consecutive mono-intercalation steps, characterised by two distinct equilibrium constants (with the average number of base pair per binding site equals 3.5): $K_{1}\;=\;3.36\;\pm \;0.43\;\times \;10^{7}\;M^{-1}$ and $K_{2}=1.90\;\pm \;0.61\;\times \;10^{5}\;M^{-1}$, respectively. Mono-intercalation dominates at high concentrations of YOYO-1. Bis-intercalation occurs at low concentrations.

## 1. Introduction

The reactions in living cells occur in nanomolar or even picomolar concentrations. Quantitative analysis of reactions at such small concentrations requires understanding the reaction mechanism and appropriate methods to determine the equilibrium constants, *K*. In solutions, only fluorescence techniques provide sufficient sensitivity at the nanomolar concentrations scale. The methods include fluorescence correlation spectroscopy (FCS) [1], fluorescence resonance energy transfer (FRET) [2], total internal reflection fluorescence microscopy (TIRF) [3], and protein-induced fluorescence enhancement (PIFE) [4]. Recently, we developed a method for precise and accurate determination of equilibrium constants of biochemical complex formations at the subnanomolar concentration range [5]. The method is based on a change of intrinsic molecular brightness (MB) of fluorescent molecules upon complex formation. Our brightness analysis method (BAM) is applicable even for reactions characterised by changes in MB as small as 5% [6]. We use this method to study DNA staining, providing a much higher sensitivity than microscopic imaging, which requires a submicromolar-micromolar dye concentration regime.

**Figure 1:**
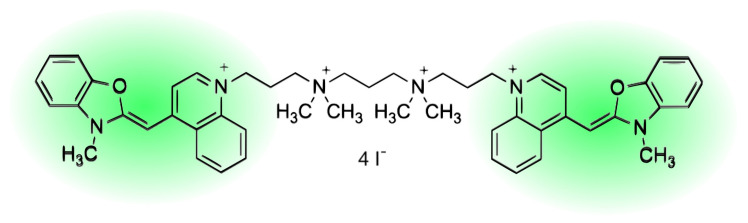
Structure of YOYO-1 molecule. Fluorescent groups of the molecule marked with green areas.

**Figure 2:**
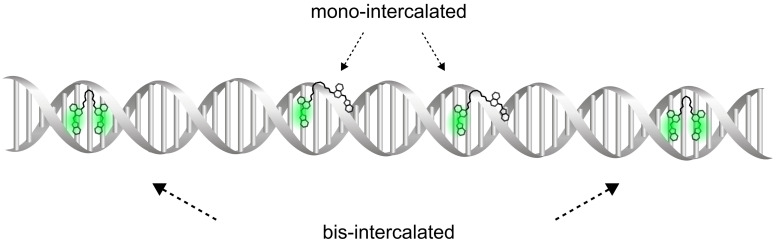
Scheme of two binding models of YOYO-1 to DNA. The complex can be formed by intercalating mer of YOYO-1 molecule to DNA, while the second mer is electrostatically bound along the helix (mono-intercalated YOYO-1) or intercalated (bis-intercalated YOYO-1). Different molecular brightnesses characterise each binding mechanisms.

The cyanine dyes create complexes with nucleotides, enabling the labelling of target DNA strands, their detection, and size determination [7,8,9,10]. One of the representatives is commonly used YOYO-1 dye (1,1’-(4,4,7,7-tetramethyl-4,7-diazaundecamethylene)-bis-4-[3-methyl-2,3-dihydro-(benzo-1,3-oxazole)-2-methylidene]-quinolinium tetraiodide) [11] (Figure 1). The dye increases its quantum yield 1000-fold upon intercalation into dsDNA [12]. Since the dye is binding to dsDNA, and is widely applied to DNA study [7,8,13,14]. Although the dye has been used for thirty years, the mechanism of binding and its strength is still not defined. YOYO-1 dye consists of the two mers on a linker (Figure 1). Each mer can intercalate into DNA. The linker is long enough to allow the intercalation of the two mers into the DNA molecule together (Figure 2). Therefore, we consider two reactions. The first one occurs when one mer of YOYO-1 dye binds to DNA, creating mono-intercalated YOYO-1 complex (DNA−YOYO_m_), with the equilibrium constant, $K_{1}$.

$$\begin{array}{c} \mathrm{DNA}+\mathrm{YOYO}\overset{K_{1}}{⇌}\mathrm{DNA}-{\mathrm{YOYO}}_{m} \end{array}$$

The second reaction occurs when the linker bends, allowing the second mer to intercalate into DNA (DNA−YOYO_b_), with the equilibrium constant, $K_{2}$:

$$\begin{array}{c} \mathrm{DNA}+\mathrm{DNA}-{\mathrm{YOYO}}_{m}\overset{K_{2}}{⇌}\mathrm{DNA}-{\mathrm{YOYO}}_{b} \end{array}$$

The available reports regarding the equilibrium constant data, although suggested the two-mode DNA-YOYO-1 binding, provides only the K values for bis-intercalation mode [9,10,15,16]. Moreover, the data exhibit significant differences spanning over three orders of magnitude (see Table 1).

Here, we prove the robustness of our method in a study on DNA-YOYO-1 complex formation. The analysis of MB change upon YOYO-1 binding to the DNA provides two-step intercalation mechanisms and determine respective equilibrium constants.

## 2. Results

### 2.1. The Validation of DNA-YOYO-1 Binding Models

**Figure 3:**
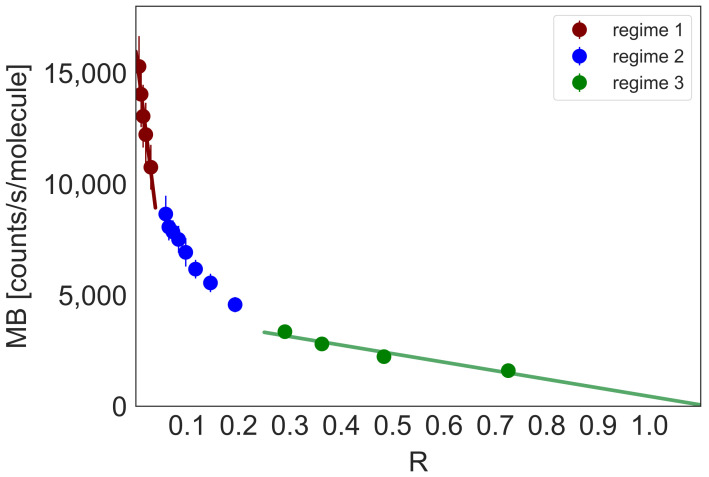
The changes of the average molecular brightness (MB) of the YOYO-1 upon the reaction with the DNA (69 bp) plotted as a function of the ratio of YOYO-1 to DNA in bp concentrations. Depending on R, we can distinguish three different trends of the MB change. Regime 1 corresponds to the bis-intercalated YOYO-1 complex, regime 3 to the mono-intercalated YOYO-1. In regime 2, both intercalation reactions occur. The fraction of each mono and bis-intercalated dye changes with R.

We performed titration experiments, changing DNA bp concentration with constant dye concentration (20 nM) to explore the DNA-YOYO-1 binding models. We monitored the MB change of DNA-YOYO-1 complex depending on YOYO-1 to DNA concentrations ratio ($R\;=\;C_{\mathrm{YOYO}-1}/C_{\mathrm{DNA}}$), as presented in Figure 3. We observed three regimes. In the first regime (R < 0.03), only bis-intercalation occurs.

For the ratio R > 0.29, we define the regime in which only one mer of YOYO-1 binds to the DNA strand. Between the two regimes, the bis-intercalation reaction competes with the non-specific binding to phosphate DNA groups (see Supplementary Materials, section Experiment with constant DNA concentration).

### 2.2. Equilibrium Constants of DNA-YOYO-1 Complexes Formation

**Figure 4:**
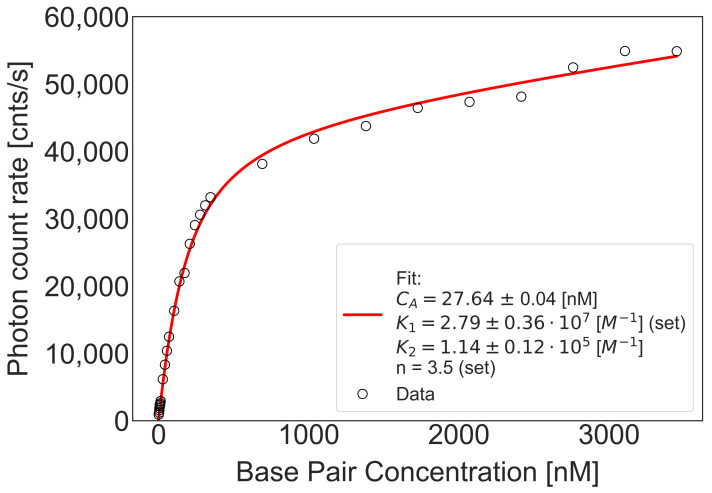
Equilibrium constant determination of YOYO-1–DNA (69 bp) complex formation. Black circles represent data points (countrate acquired for various DNA concentrations added to 20 nM YOYO-1 in titration experiment), red line correspond to fit of Equation (7). Fitting includes interactions for both intercalation models. $C_{A}$ is a fitted value of dye initial concentration, $K_{1}$ and $K_{2}$ are equilibrium constants for mono-intercalation and bis-intercalation, respectively, n is a size of a binding site (see Supplementary Materials, section Size of the binding site).

We found that DNA-YOYO-1 complex formation is a two steps reaction. We determined equilibrium constants for both steps of reaction, mono-intercalation and bis-intercalation of YOYO-1. In a series of titration experiments, we varied DNA concentration and kept YOYO-1 concentration constant (20 nM). In each titration point, we measured count rate and plotted it as a function of DNA in bp concentration (Figure 4). We used calculated molecular brightness (see Materials and Method) and Equation (5) to calculate $K_{1}$ (for R>0.29) (see Supplementary Materials, section Mono-intercalation equilibrium constant); $K_{1}$ and $C_{\mathrm{YOYO}-1}^{0}$ were used as free parameters. Next, using above-calculated $K_{1}$, we fitted data in whole range of concentrations to obtain $K_{2}$ with Equation (7). Obtained mean values of equilibrium constants are equal to: $K_{1}\;=\;3.36\;\pm \;0.43\;\times \;10^{7}\;M^{-1}$ and $K_{2}=1.90\;\pm \;0.61\;\times \;10^{5}\;M^{-1}$. Obtained $K_{1}$ and $K_{2}$ values suggests that the formation of the mono-intercalated complex is about 200 times more preferable than bis-intercalation of YOYO-1 dye. Our results confirm and quantify two-mode mechanism suggested in previous reports [9,10,15,16].

## 3. Discussion

The numerous studies on the mechanism of YOYO-1 intercalation into the DNA have found the equilibrium constant only for bis-intercalation [17,18]; as we summarised in Table 1. However, the determined values differ in 3 orders of magnitude ($10^{6}-10^{9}$). In comparison to these reports, we demonstrate that binding of YOYO-1 to the DNA occurs with two modes of reaction characterised by distinct equilibrium constants.

The two modes of reaction were suggested in a few studies conducted by different methods. The first study was linear and circular dichroism made by Larsson et al. [15]. However, this spectral analysis did not give the equilibrium constant of the reaction. Other studies published by Murade et al. utilised single-molecule microscopy and optical tweezers aimed at describing the force-extension of DNA in the presence of YOYO-1 and its monomer YO-PRO [9]. The observed differences in the kinetics of YOYO-DNA and YO-DNA interaction during stretching DNA was explained by bis-intercalation occurring in two steps. The proposed kinetic model was not supported by reaction rates values nor equilibrium constants. The two-mode reaction was also proposed by Paik et al. [16]. Force-enhanced intercalation of YOYO-1 into the DNA in the presence of monovalent salts at different concentration revealed two binding states of YOYO-1. At low salts concentration, YOYO-1 was assumed to be bis-intercalated, while at high salt concentration YOYO-1 was mono-intercalated or bound only electrostatically. Similar interaction of YOYO-1 with DNA was observed by Wang et al. using magnetic tweezers [10]. As an outcome, the equilibrium constant was determined, however, only for one mode of the reaction. In any of the mentioned reports, two modes of intercalation were not characterised by respective equilibrium constants. Furthermore, the mentioned reports were based on methods involving the change of DNA conformation. The external forces (force spectroscopy or magnetic tweezers) applied to DNA might influence the equilibrium constant value.

Here, our non-invasive brightness analysis to study YOYO-1 intercalation into the DNA not only validate two modes of reaction but also quantified the respective equilibrium constants.

## 4. Materials and Methods

### 4.1. Materials

We used double-stranded DNA (69 bp, see Supplementary Materials, section DNA sequence) synthesised by IBA GmbH, Göttingen, Germany. DNA was stored in standard Tris-EDTA buffer (Sigma-Aldrich, St. Louis, MO, USA) at −20 ${}^{\circ }$C. Prior to the measurements, DNA was diluted in a series of working concentrations from 20 pM to 50 nM. The solvent was phosphate buffer saline, PBS (pH = 7.4, a phosphate buffer concentration of 0.01 mM, 0.0027 M potassium chloride and 0.137 M sodium chloride) with the addition of 0.002% Tween20 surfactant. The surfactant was used to avoid absorption of the compounds on the sample chamber walls, which would preclude quantitative concentration measurements [19]. YOYO-1 dye (Invitrogen, Waltham, MA, USA) was added to the DNA solution 2 h before the fluorescence correlation spectroscopy (FCS) measurements to a final concentration of 20 nM.

### 4.2. FCS Set-Up

The FCS set-up included the Nikon EZ-145 C1 microscope (Nikon, Tokyo, Japan) integrated with time-correlated single-photon counting (TCSPC) data acquisition system (PicoHarp 300, PicoQuant, Berlin, Germany). All measurements were carried out using Nikon PlanApo 60x water immersion objective (NA = 1.2). Excitation was done using the laser diode emitting picosecond pulses with a wavelength of 485 ± 3 nm. The signal was recorded by detector based on Single Photon Avalanche Diode (SPAD) (PerkinElmer Optoelectronics, Wiesbaden, Germany). The fluorescence was detected through a 488 long-pass filter (Chroma, Brattleboro, VT, USA), positioned in the optical path in front of the detector. Data acquisition was performed using Symphotime 64 software (PicoQuant, Berlin, Germany). For temperature control, we used the Okolab Cage Incubation System (Okolab, Puzzuoli, Italy), which stabilises the temperature with the accuracy of $0.5{\;}^{\circ }$C.

### 4.3. FCS Measurements

The temperature was set at $25\pm 0.5{\;}^{\circ }C$. We used laser power of 47µW (measured before the objective by power meter PM400, Thorlabs, Newton, NJ, USA). At such low laser power, there was no photobleaching of molecules, and the contribution of triplet state in acquired signal was reduced (see Supplementary Materials, section YOYO-1 dye). The experiments were preceded by establishing the confocal volume dimensions using calibration for rhodamine 110 (Sigma-Aldrich, St. Louis, MO, USA) dissolved in PBS (see Supplementary Materials, section Calibration of the FCS set-up). For all measurements, we used the glass-bottom container (µ-Slide 8 Well Glass Bottom, Ibidi, Martinsried, Germany). FCS measurements were performed 10µm above the glass cover. Acquired data were analysed by using self-written Python scripts.

### 4.4. Brightness Method

**Figure 5:**
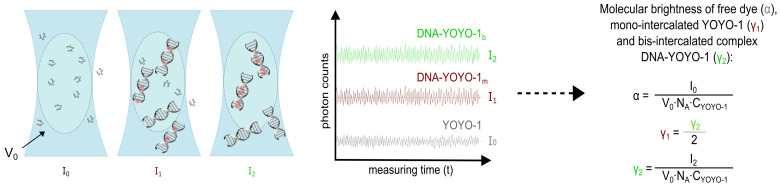
Scheme of the method of molecular brightness determination. In the confocal volume ($V_{0}$) we collect photons in time (t) by the detector. Knowing the intensity of free and bound dye ($I_{0}$, $I_{1}$ and $I_{2}$) we determine MB for free dye (α = 28 counts per molecule per second, cpm), mono-intercalated YOYO-1 ($\gamma_{1}$ = 8400 cpm) and bis-intercalated complex ($\gamma_{2}$ = 16,800 cpm), respectively.

We utilised the brightness method to study the mechanism of the DNA-YOYO-1 binding reaction. The technique involves the change of molecular brightness of reactants upon complex formation. The weakly fluorescent YOYO-1 binds to DNA and forms a bright fluorescent complex. We monitored the fluorescence intensity of the YOYO-1 dye (free and intercalated) to determine the MB, which is defined as the number of emitted photons per molecule per second. The molecular brightness of the free dye (α) can be determined directly from the fluorescence intensity measurements:

(1) $$I_{0}=V_{0}\cdot N_{A}\cdot C_{\mathrm{YOYO}}\cdot \alpha$$

where $V_{0}$ is focal volume measured during calibration, $N_{A}$ is the Avogadro number, $C_{\mathrm{YOYO}}$ is the dye concentration, and $I_{0}$ is a fluorescence intensity of the free dye (measured in the same experimental conditions in the absence of DNA). Due to the extremely weak fluorescence of the unbound YOYO-1 (see Supplementary Materials, section YOYO-1 dye), we used 1µM dye solutions to measure alpha. At μM concentrations, the fluctuations in the number of molecules in the focal volume are negligible. Therefore, the mean number of molecules in focal volume is constant and equals $N_{\mathrm{YOYO}-1}\;=\;C_{\mathrm{YOYO}-1}\;\cdot \;N_{A}\;\cdot \;V_{0}$ (Figure 5).

For mono-intercalation reaction, the equilibrium constant $K_{1}$ is given by:

(2) $$K_{1}\;=\;\frac{C_{\mathrm{DNA}-{\mathrm{YOYO}}_{m}}^{\mathrm{eq}}}{C_{\mathrm{YOYO}}^{\mathrm{eq}}\cdot C_{\mathrm{DNA}}^{\mathrm{eq}}}$$

For bis-intercalation reaction, the equilibrium constant $K_{2}$ equals to:

(3) $$K_{2}\;=\;\frac{C_{\mathrm{DNA}-{\mathrm{YOYO}}_{b}}^{\mathrm{eq}}}{C_{\mathrm{DNA}-{\mathrm{YOYO}}_{m}}^{\mathrm{eq}}\cdot C_{\mathrm{DNA}}^{\mathrm{eq}}}$$

where $C_{\mathrm{YOYO}-1}^{\mathrm{eq}}$, $C_{\mathrm{DNA}}^{\mathrm{eq}}$, $C_{\mathrm{DNA}-\mathrm{YOYO}-1_{m}}^{\mathrm{eq}}$ and $C_{\mathrm{DNA}--1_{b}}^{\mathrm{eq}}$ are concentrations in equilibrium state of free dye, DNA, mono-intercalated and bis-intercalated complex, respectively. The intensity of fluorescence for mono-intercalated YOYO-1 ($I_{1}$) complex is given by:

(4) $$I_{1}=V_{0}\cdot N_{A}\cdot \left(C_{\mathrm{YOYO}}^{\mathrm{eq}}\cdot \alpha +C_{\mathrm{DNA}-{\mathrm{YOYO}}_{m}}^{\mathrm{eq}}\cdot \gamma_{1}\right)$$

where $\gamma_{1}$ is MB of mono-intercalated YOYO-1 complex with DNA. Using equilibrium constant $K_{1}$, we solved the quadratic equation for $C_{\mathrm{DNA}-{\mathrm{YOYO}}_{m}}^{\mathrm{eq}}$ (see Supplementary Materials, section Brightness Method). We rewrote Equation (4), replacing complex concentration with the quadratic equation’s solution (Equation (2)):

(5) $$I_{1}=V_{0}\cdot N_{A}\cdot \;\alpha \cdot \;\left(\left(C_{\mathrm{YOYO}}^{0}-C_{\mathrm{DNA}-{\mathrm{YOYO}}_{m}}^{\mathrm{eq}}\right)\;+\left(\frac{\gamma_{1}}{\alpha }\cdot K_{1}\;\cdot \;\left(C_{\mathrm{YOYO}}^{0}-C_{\mathrm{DNA}-{\mathrm{YOYO}}_{m}}^{\mathrm{eq}}\right)\;\cdot \left(C_{\mathrm{DNA}}^{0}-C_{\mathrm{DNA}-{\mathrm{YOYO}}_{m}}^{\mathrm{eq}}\right)\right)\right)$$

Analogically, we wrote the following equations for bis-intercalation. Firstly, for measured intensity (Equation (6)). We used Equation (3) to derive bis-intercalated YOYO-1 concentration and rewrote the equation, relating it to respective equilibrium constants (Equation (7)).

(6) $$I_{2}=V_{0}\cdot N_{A}\cdot \left(C_{\mathrm{YOYO}}^{\mathrm{eq}}\cdot \alpha +C_{\mathrm{DNA}-{\mathrm{YOYO}}_{m}}^{\mathrm{eq}}\cdot \;\gamma_{1}\;+\;C_{\mathrm{DNA}-{\mathrm{YOYO}}_{b}}^{\mathrm{eq}}\cdot \;\gamma_{2}\right)$$

(7) $$\begin{array}{c} I_{2}=V_{0}\cdot N_{A}\cdot \;\alpha \cdot \;(\left(C_{\mathrm{YOYO}}^{0}-C_{\mathrm{DNA}-{\mathrm{YOYO}}_{m}}^{\mathrm{eq}}\right)\;+\left(\frac{\gamma_{1}}{\alpha }\cdot K_{1}\;\cdot \;\left(C_{\mathrm{YOYO}}^{0}-C_{\mathrm{DNA}-{\mathrm{YOYO}}_{m}}^{\mathrm{eq}}\right)\;\cdot \left(C_{\mathrm{DNA}}^{0}-C_{\mathrm{DNA}-{\mathrm{YOYO}}_{m}}^{\mathrm{eq}}\right)\right)\\ +\;(\frac{\gamma_{2}}{\alpha }\cdot K_{2}\cdot \;C_{\mathrm{DNA}-{\mathrm{YOYO}}_{m}}^{\mathrm{eq}}\;\cdot \;\left(C_{\mathrm{DNA}}^{0}-C_{\mathrm{DNA}-{\mathrm{YOYO}}_{m}}^{\mathrm{eq}}\right))) \end{array}$$

where $\gamma_{2}$ is the intrinsic molecular brightness of the bis-intercalated YOYO-1 complex. We determined $\gamma_{2}$ in an experiment with the excess of DNA (one molecule of YOYO-1 per one DNA molecule), as shown in Figure 5. Since YOYO-1 is a dimer molecule, the MB of mono-intercalated YOYO-1 ($\gamma_{1}$) is half of the bis-intercalated dye ($\gamma_{2}$).

## 5. Conclusions

We found brightness method as accurate for determination of equilibrium constants for YOYO-1 intercalation into DNA structure. We obtained equilibrium constants for both presented binding modes of the dye, $K_{1}\;=\;3.36\;\pm \;0.43\;\times \;10^{7}\;M^{-1}$ for mono-intercalated complex and $K_{2}=1.90\;\pm \;0.61\;\times \;10^{5}\;M^{-1}$ for bis-intercalated complex. The method was sensitive enough to determine intrinsic MB, despite the weak fluorescent properties of free dye. Change of MB resulted in understanding and characterising two models of intercalation. Mode of the interactions depends on the ratio of dyes molecules to DNA base pairs concentration (R). We observed three regimes of DNA concentration, for bis-intercalation only (R < 0.03), for mono-intercalated YOYO-1 complex only (R > 0.29), and the regime between the two, with both modes present. We have confirmed the accuracy of our brightness method. We believe that this noninvasive and sensitive method will serve as a base for future studies on a biochemical reactions in a complex system, i.e., the interior of the living cells. 

## Figures and Tables

**Table 1:** Literature values of equilibrium constant for bis-intercalation DNA-YOYO-1 complex formation determined by different methods.

Method	$K\;[M^{-1}]$
Magnetic tweezers [10]	$3.58\times 10^{6}$
Fluorescence spectroscopy [17]	$4.6\times 10^{6}$
Force extension fitting [18]	$1.4\times 10^{9}$

## Data Availability

The data presented in this study are available on request from the corresponding author.

## Supplementary Materials

The supplementary materials are available online. References [5,10,18] are cited in the supplementary materials.

## Sample Availability

Samples of the compounds are not available from the authors.
