# Supplementary material for: Two Intercalation Mechanisms of Oxazole Yellow Dimer (YOYO-1) into DNA
Source: Molecules. 2021 Jun 19;26(12):3748. doi: 10.3390/molecules26123748 (PMC8234192; doi:10.3390/molecules26123748)
Supplement: Supplementary file 1 [file molecules-26-03748-s001.zip › molecules-1253971-supplementary-proofread/Holyst_support.pdf]

Supplementary Materials: Two Intercalation Mechanisms of Oxazole Yellow Dimer (YOYO-1) into DNA

Karolina Kucharska, Marta Pilz, Krzysztof Bielec, Tomasz Kalwarczyk, Patrycja Kuźma and Robert Hołyst *

Table of Content:

S1 DNA sequence 2

S2 Calibration of the FCS set-up 2

S3 YOYO-1 dye 3

S4 Experiment with constant DNA concentration 4

S5 Brightness model 4

S6 Size of the binding site 6

S7 Mono-intercalation equilibrium constant 8

References 9

S1. DNA sequence

In the experiments, we used a 69 bp double-stranded oligonucleotide, synthesised by IBA GmbH, Germany. The DNA sequence is:

5′ GAT ACG AGC ATC GTG TAG GCA TCG TAG GTA ATA CGG ATG TGC TAG (...)
3′ CTA TGC TCG TAG CAC ATC CGT AGC ATC CAT TAT GCC TAC ACG ATC (...)

(...) CTT ATT GAA TTC AGA GAT CTA TGC 3′
(...) GAA TAA CTT AAG TCT CTA GAT ACG 5′

S2. Calibration of the FCS set-up

We calibrated the FCS set-up prior measurements, utilising fluorescent dye with known size and diffusion coefficient. We used rhodamine 110 (470 D = $\mu m^2/s$) at temperature applied in measurements (25 °C) to determine semi-minor axis of the confocal volume (ω), and the effective volume (V_{eff}). We performed 28 separate measurements of the dye and fitted the data with a one-component autocorrelation function, G(t) (Eqn S1).

$$G(t) = \left(\frac{1}{N}\right) \cdot \left(1 + \frac{T}{1+T} \cdot \left(\frac{1}{\exp\frac{t}{\tau_{triplet}}}\right)\right) \cdot \left(\frac{1}{(1+\frac{t}{\tau})}\right) \cdot \left(\frac{1}{\sqrt{1+(\frac{t}{\tau}) \cdot \frac{1}{\varkappa^2}}}\right) \quad \text{(S1)}$$

where N is the number of molecules in focal volume, T is the fraction of triplet states, $\tau_{triplet}$ is triplet lifetime, τ is the dye's time of flight through focal volume, $\varkappa$ is the aspect ratio of the focal volume.

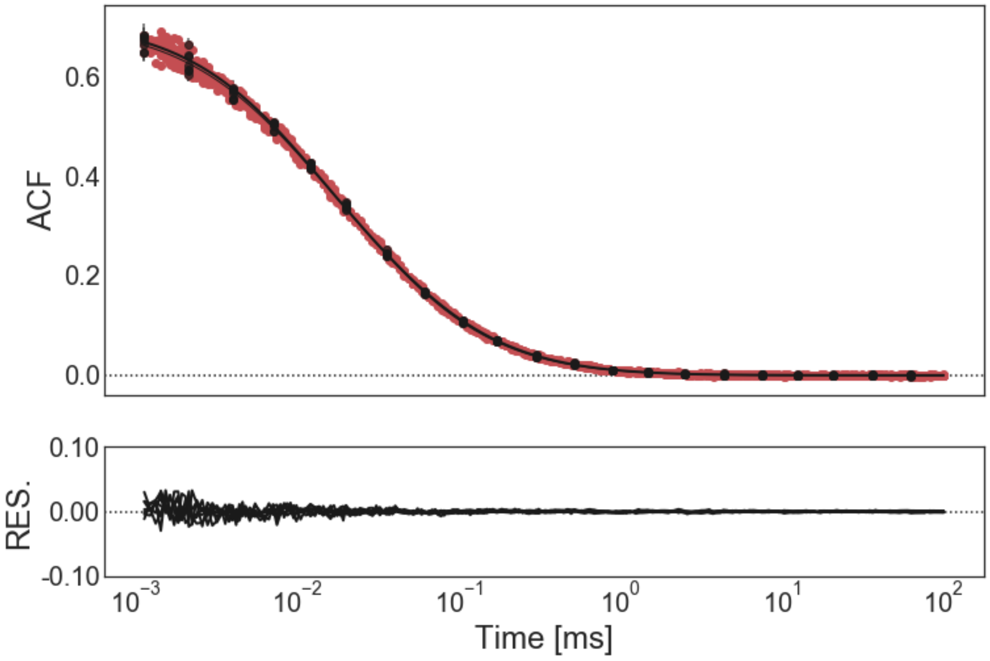

Figure S1. Example of the FCS autocorrelation curves obtained in the calibration. The measuring time for each autocorrelation curve was 20 s. The data were fitted with a one-component diffusion model including the triplet state contribution (Eqn S1). Red points represent experimental data, and the black line is the autocorrelation fitted function. The bottom panel shows the residual curve (the difference between experimental and fitted data).

The autocorrelation functions were exported to the .csv file and further analysed with self-written Python script using an appropriate diffusion model based on Equation S1 with nonlinear least square fitting method (Fig. Supplementary S1); N, T, $\tau_{triplet}$, τ and $\varkappa$ were used as free parameters.

The obtained values from measurements were averaged, and the semi-minor axis of the confocal volume, ω was calculated using the formula:

$$\omega = \sqrt{4\tau D} \tag{S2}$$

Then, the determined value of omega was used to calculate the effective volume of the confocal focus, V_{eff}:

$$V_{eff} = \pi^{3/2}\varkappa\omega^3 \tag{S3}$$

S3. YOYO-1 dye

Free YOYO-1 has a low quantum yield. We measured YOYO-1 dye in a wide range of laser powers (Fig. Supplementary S2) and concentrations (Table Supplementary S1) to calculate its intrinsic molecular brightness. The increase in laser power does not influence the dye's fluorescence properties. We do not detect autocorrelation function in any of the used laser powers. We performed experiments using various dye concentration to distinguish the sample and background signals properly and calculated the molecular brightness of the free dye, α.

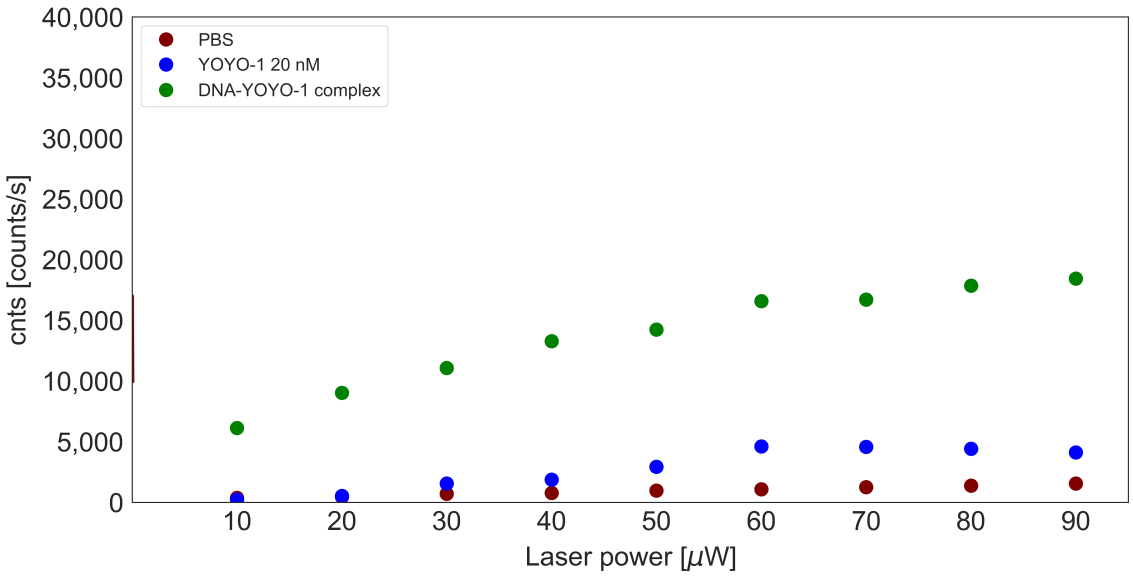

Figure S2. Response of the samples after excitation with different laser power. PBS and YOYO-1 dye do not show fluorescence; the collected signal comes from the dispersion of the laser beam on molecules. The complex of DNA (2 nM) and YOYO-1 (20 nM) exhibit fluorescence properties. Response from the sample is linear in the laser power below 50 μW. For higher powers, the complex is photo-bleaching during the measurement, and the response of the sample is not linear.

Table S1. Collected photons from the YOYO-1 samples at various concentrations; laser power: 47 μW.

Concentration of YOYO-1	count rate
1μM	5848
500 nM	4700
100 nM	3612
50 nM	2888
10 nM	1506
5 nM	1420
1 nM	975
0.5 nM	929

S4. Experiment with constant DNA concentration

We performed experiments with a constant DNA concentration (5 nM), varying concentration of YOYO-1 dye. DNA at 5 nM concentration allowed us to study a single DNA strand in the focal volume. The increase of the dye concentration increases the number of collected photons. The photon count rate reached the plateau for YOYO-1 concentration of 75 nM and higher (Fig. Supplementary S3). In the plateau concentrations regime, the dye saturated DNA strand. In high dye concentrations mono-intercalated complex of YOYO-1 dominates. Dividing the photon count rate from the plateau region by brightness of the mono-intercalated complex (γ_1 = 8400 cpm), we assumed that 8 molecules of YOYO-1 are mono-intercalated in a single DNA strand.

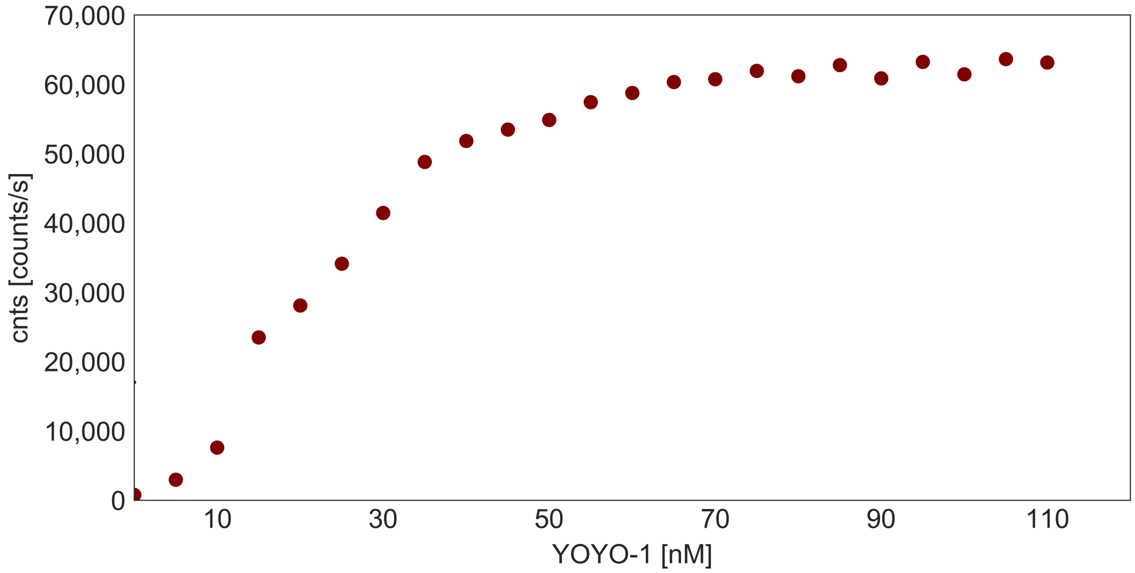

Figure S3. Collected photons displayed as a function of YOYO-1 concentration. The dye created a complex with DNA (5 nM), resulting in an increase of detected photons with increasing dye concentration. Saturation of the DNA strand was achieved at 75 nM of the dye.

S5. Brightness model

In the brightness analysis method, we monitor the change in molecular brightness (MB) upon occurring reaction, here, complex formation. MB is defined as a number of photons ($N_{photons}$) emitted by a molecule in time (t). It depends on fluorescence molecule properties, laser power, wavelength and sensitivity of the detectors [1]. MB is given by:

$$MB = \frac{1}{t}\frac{N_{photons}}{N_{molecules}} \tag{S4}$$

where $N_{molecules}$ is the number of molecules in the focal volume. The number of emitted photons in time is described by the count rate (I):

$$I = \frac{N_{photons}}{t} \tag{S5}$$

Using equation S5, we rewritten MB:

$$MB = \frac{I}{N_{molecules}} \tag{S6}$$

We expressed the number of molecules in terms of concentrations, using the Avogadro constant (N_A) and focal volume (V_{eff}):

$$N_{molecules} = C \cdot N_A \cdot V_{eff} \tag{S7}$$

Combining equations S6 and S7, the count rate equals to:

$$I = MB \cdot C \cdot N_A \cdot V_{eff} \tag{S8}$$

Intensity (I_0) of the free dye, characterised by its molecular brightness (α), is given by:

$$I_0 = \alpha \cdot C_{YOYO-1} \cdot N_A \cdot V_{eff} \tag{S9}$$

where C_{YOYO-1} is the concentration of the free dye. After the addition of DNA, the complex formation occurs. In excess of the DNA, bis-intercalation dominates, while in excess of the dye, YOYO-1 creates a mono-intercalated complex ($DNA - YOYO_m$). Moreover, bis-intercalation consists of two mono-intercalations. Mono-intercalation is described with the reaction:

$$DNA + YOYO \xrightleftharpoons{K_1} DNA\text{–}YOYO_m$$

Equilibrium constant, K_1, for that reaction is given by:

$$K_1 = \frac{C^{eq}_{DNA_{YOYO_m}}}{C^{eq}_{DNA}C^{eq}_{YOYO-1}} = \frac{C^{eq}_{DNA_{YOYO_m}}}{(C^0_{DNA} - C^{eq}_{DNA_{YOYO_m}})(C^0_{YOYO-1} - C^{eq}_{DNA_{YOYO_m}})} \tag{S10}$$

where $C^{eq}_{DNA_{YOYO_m}}$ is the concentration of mono-intercalated complex in the equilibrium state, C^0_{YOYO-1} and C^0_{DNA} are initial concentrations of the dye and the DNA, respectively. We wrote a quadratic equation with the concentration of mono-intercalated complex in equilibrium state as a variable:

$$K_1(C^{eq}_{DNA_{YOYO_m}})^2 - C^{eq}_{DNA_{YOYO_m}}(K_1C^0_{DNA} + K_1C^0_{YOYO-1} + 1) + K_1C^0_{DNA}C^0_{YOYO-1} = 0 \tag{S11}$$

We solved equation S11, obtaining the relation describing the concentration of the mono-intercalated complex in equilibrium state:

$$C^{eq}_{DNA_{YOYO_m}} = \frac{1}{2} \cdot (C^0_{DNA} + C^0_{YOYO-1} + \frac{1}{K_1} - \sqrt{(-C^0_{DNA} - C^0_{YOYO-1} - \frac{1}{K_1})^2 - 4C^0_{DNA}C^0_{YOYO-1}}\,) \tag{S12}$$

We describe intensity (I_1) for the probe with mono-intercalated complex, as:

$$I_1 = V_{eff} \cdot N_A \cdot (\alpha \cdot C^{eq}_{YOYO-1} + \gamma_1 \cdot C^{eq}_{DNA_{YOYO_m}}\,) \tag{S13}$$

where γ_1 is MB of the mono-intercalated complex. We combined equations S10 and S13, to relate intensity with initial concentrations and equilibrium constant, K_1:

$$I_1 = V_{eff} \cdot N_A \cdot \alpha \cdot ((C^0_{YOYO-1} - C^{eq}_{DNA_{YOYO_m}}) + \frac{\gamma_1}{\alpha} \cdot K_1 \cdot (C^0_{DNA} - C^{eq}_{DNA_{YOYO_m}}) \cdot (C^0_{YOYO-1} - C^{eq}_{DNA_{YOYO_m}})) \tag{S14}$$

We incorporated size of the binding site into the model by describing DNA concentration as the number of binding sites:

$$I_1 = V_{eff} \cdot N_A \cdot \alpha \cdot ((C^0_{YOYO-1} - C^{eq}_{DNA_{YOYO_m}}) + \frac{\gamma_1}{\alpha} \cdot K_1 \cdot (\frac{C^0_{DNA}}{n} - C^{eq}_{DNA_{YOYO_m}}) \cdot (C^0_{YOYO-1} - C^{eq}_{DNA_{YOYO_m}})) \tag{S15}$$

We described bis-intercalation reaction with the equation:

$$DNA + DNA{-}YOYO_m \xrightleftharpoons{K_2} DNA{-}YOYO_b$$

where $DNA - YOYO_b$ is the bis-intercalated complex. Equilibrium constant, K_2, for this reaction is given by:

$$K_2 = \frac{C^{eq}_{DNA_{YOYO_b}}}{C^{eq}_{DNA} C^{eq}_{DNA_{YOYO_m}}} \tag{S16}$$

We determined from Eqn S16 the concentration of bis-intercalated complex in equilibrium state:

$$C^{eq}_{DNA_{YOYO_b}} = K_2 \cdot (C^0_{DNA} - C^{eq}_{DNA_{YOYO_m}}) \cdot C^{eq}_{DNA_{YOYO_m}} \tag{S17}$$

Intensity (I_2) including both intercalation mechanism is given by:

$$I_2 = V_{eff} \cdot N_A \cdot (\alpha \cdot C^{eq}_{YOYO-1} + \gamma_1 \cdot C^{eq}_{DNA_{YOYO_m}} + \gamma_2 \cdot C^{eq}_{DNA_{YOYO_b}}) \tag{S18}$$

Combining Eqns S14, S17 and S18, we obtained fitting equation for bis-intercalation equilibrium constant, K_2:

$$I_2 = V_{eff} \cdot N_A \cdot \alpha \cdot ((C^0_{YOYO-1} - C^{eq}_{DNA_{YOYO_m}}) + (\frac{\gamma_1}{\alpha} \cdot K_1 \cdot (C^0_{DNA} - C^{eq}_{DNA_{YOYO_m}}) \cdot (C^0_{YOYO-1} - C^{eq}_{DNA_{YOYO_m}})) + (\frac{\gamma_2}{\alpha} \cdot K_2 \cdot (C^0_{DNA} - C^{eq}_{DNA_{YOYO_m}}) \cdot C^{eq}_{DNA_{YOYO_m}})) \tag{S19}$$

In final model for fitting K_2, DNA concentration was described as the concentration of binding sites:

$$I_2 = V_{eff} \cdot N_A \cdot \alpha \cdot ((C^0_{YOYO-1} - C^{eq}_{DNA_{YOYO_m}}) + (\frac{\gamma_1}{\alpha} \cdot K_1 \cdot (\frac{C^0_{DNA}}{n} - C^{eq}_{DNA_{YOYO_m}}) \cdot (C^0_{YOYO-1} - C^{eq}_{DNA_{YOYO_m}})) + (\frac{\gamma_2}{\alpha} \cdot K_2 \cdot (\frac{C^0_{DNA}}{n} - C^{eq}_{DNA_{YOYO_m}}) \cdot C^{eq}_{DNA_{YOYO_m}})) \tag{S20}$$

S6. Size of the binding site

Size of the binding site is one of the characteristic values for intercalators. In reports, we found inconsistency in the size of the binding site (n). The value differs between 3 and 4 base pairs per one binding site [2,3]. The size of the binding site was crucial in our analysis, as we used it in the models for fitting equilibrium constants.

We studied the binding site's size in a range of 3 to 4, varying the value by 0.1. For every n value we fitted the data with equations S15 and S20. Initial concentrations of the

DNA and the dye and molecular brightnesses of the free dye and both complexes were used as constant parameters. We analysed every set of data with all tested n values. In some cases used model did not match the fitted data (Fig. Supplementary S4). We chose n equals 3.5, based on the two conditions: firstly, all sets of data needed to be fitted by the same model, and secondly, the fitting errors had to be minimised (Fig. Supplementary S5).

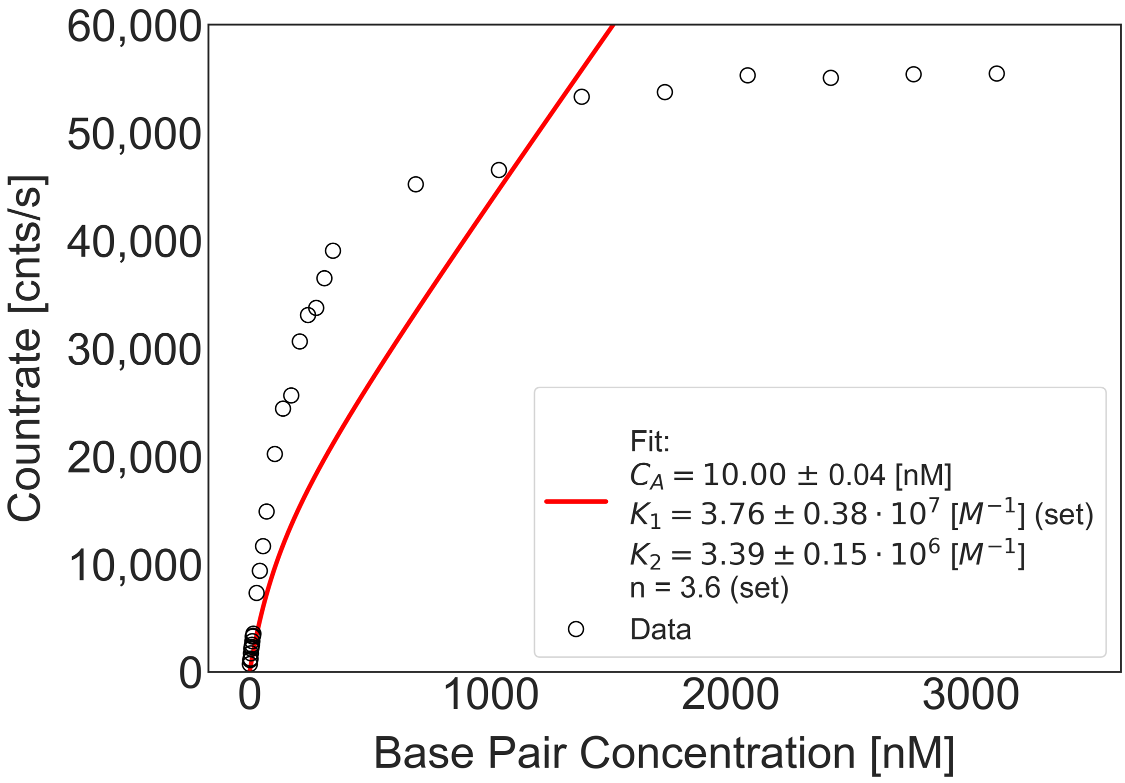

Figure S4. The fit of the equation S20 (red line) to the experimental data points (black circles). We used the binding site sizes in a range of 3 to 4, with a changing step 0.1. We used initial concentrations of the substrates, and molecular brightnesses, as constant parameters in the equation. Here, n equals 3.6, and the model used for data analysis does not fit the data. Moreover, it generates significant errors.

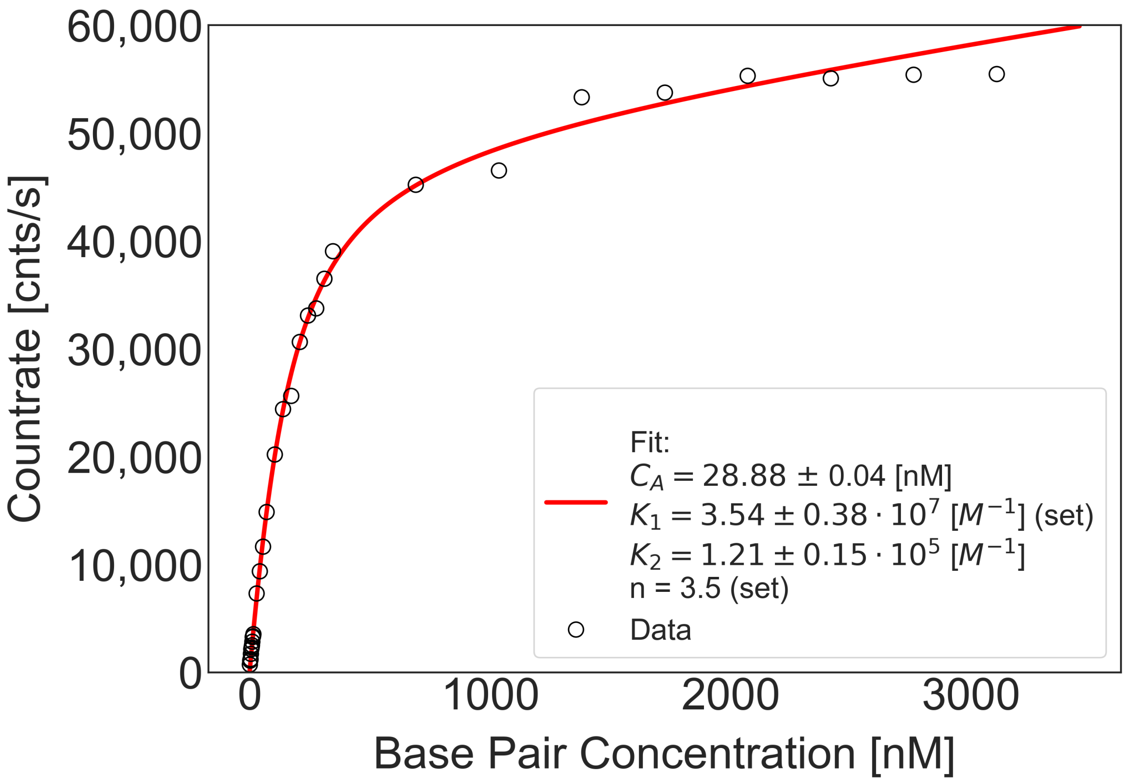

Figure S5. The fit of the equation S20 (red line) to the experimental data points (black circles). We used the binding site sizes in a range of 3 to 4, with a changing step 0.1. We used initial concentrations of the substrates, and molecular brightnesses, as constant parameters in the equation. Here, n equals 3.5. The model used for data analysis fit the data, generating errors around 10 %.

S7. Mono-intercalation equilibrium constant

We performed titration experiments in the picomolar and nanomolar concentrations range. We distinguished 3 regimes: regime 1 with bis-intercalated complexes only, regime 3 with mono-intercalated complexes only, and regime 2 with both types of the complex present (in the publication). We used the data for regime 3 to fit the equilibrium constant of mono-intercalation, K_1. We fitted K_1 with equation S14 (Fig. Supplementary S6), and used as the constant value in fitting the equilibrium constant for bis-intercalation, K_2.

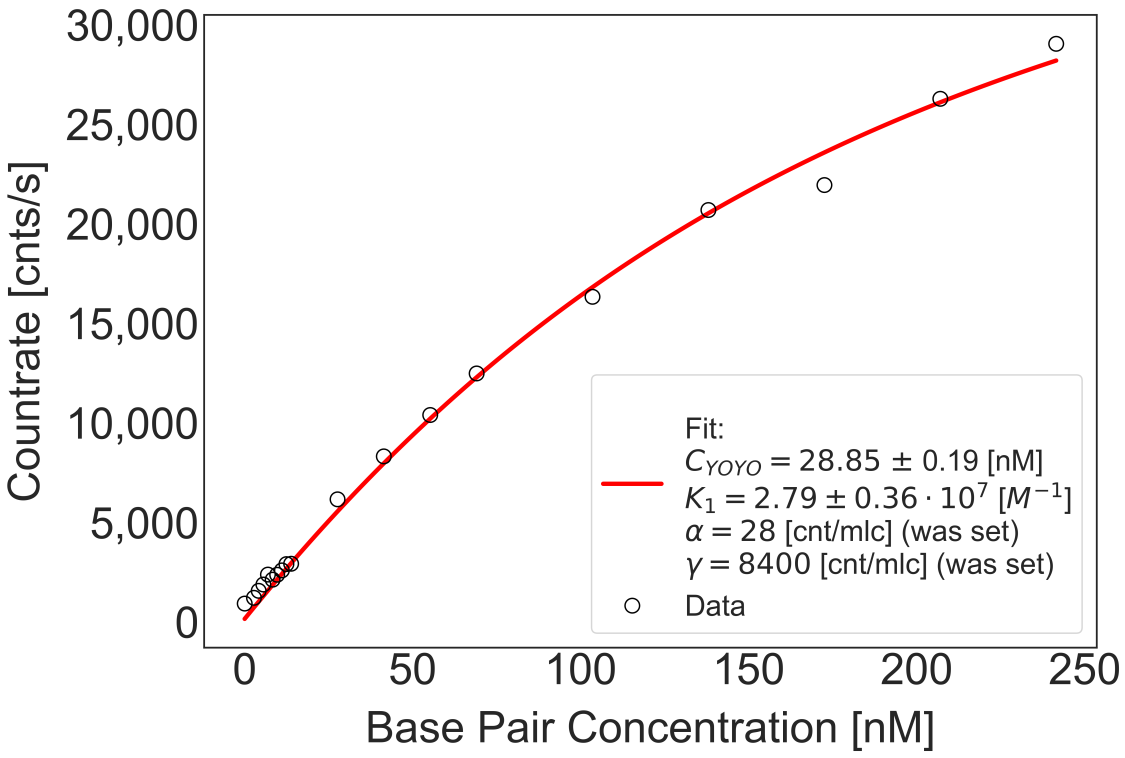

Figure S6. Equilibrium constant determination of YOYO-1–DNA (69 bp) complex formation. Black circles represent data points (countrate acquired for various DNA concentrations in a range of regime 3 added to 20 nM YOYO-1 in titration experiment), red line corresponds to the fit of Eqn S14. Fitting includes only mono-intercalation. C_A is a fitted value of dye's initial concentration, K_1 is the equilibrium constant of mono-intercalation, n is a size of a binding site.

References

1. Bielec, K.; Bubak, G.; Kalwarczyk, T. and Hołyst, R. Analysis of Brightness of a Single Fluorophore for Quantitative Characterization of Biochemical Reactions. *J. Phys. Chem. B* **2020**, *124*, 1941-1948.
2. Wang, Y.; Sischka, A.; Walhorn, V.; Tönsing, K. and Anselmetti, D. Nanomechanics of Fluorescent DNA Dyes on DNA Investigated by Magnetic Tweezers. *Biophys. J.* **2016**, *111*, 1604-1611.
3. Günter, K.; Mertig, M. and Seidel, R. Mechanical and structural properties of YOYO-1 complexed DNA. *Nucleic Acids Res.* **2010**, *38*, 6526-6532.
