# Supplementary figures and images for: Two Intercalation Mechanisms of Oxazole Yellow Dimer (YOYO-1) into DNA
Source: Molecules. 2021 Jun 19;26(12):3748. doi: 10.3390/molecules26123748 (PMC8234192; doi:10.3390/molecules26123748)

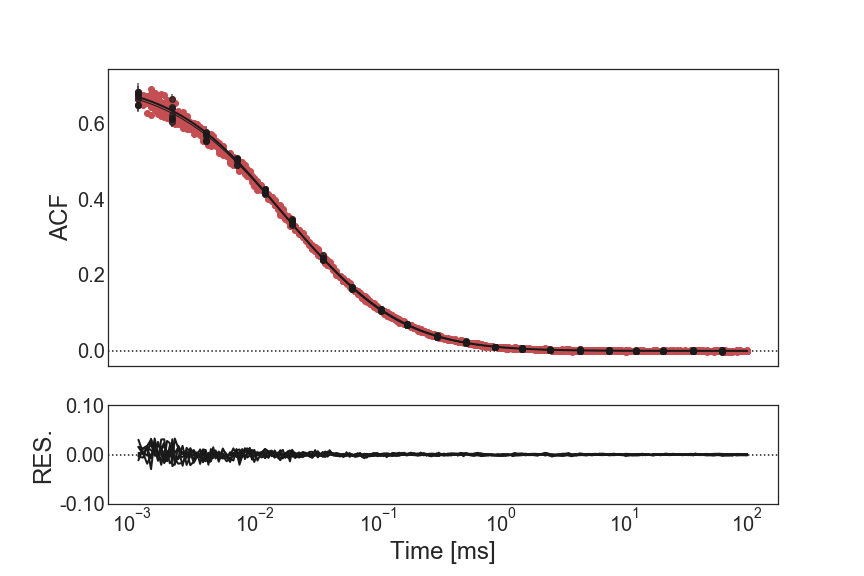

Supplement: Supplementary file 1 [file molecules-26-03748-s001.zip › molecules-1253971-supplementary-proofread/cal.png]

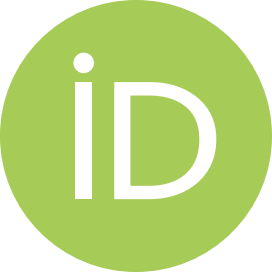

Supplement: Supplementary file 1 [file molecules-26-03748-s001.zip › molecules-1253971-supplementary-proofread/Definitions/logo-orcid-eps-converted-to.pdf]

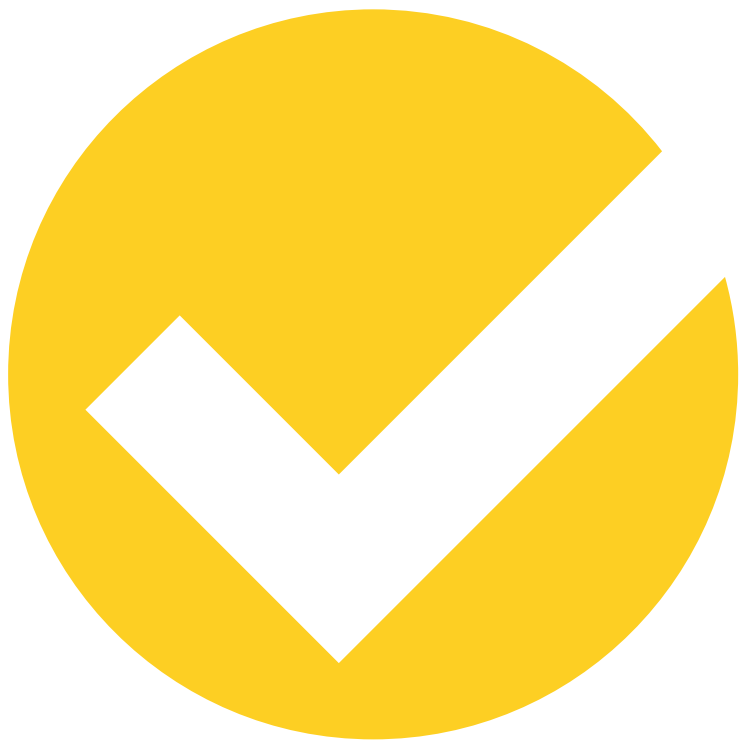

check for  
updates

Supplement: Supplementary file 1 [file molecules-26-03748-s001.zip › molecules-1253971-supplementary-proofread/Definitions/logo-updates.pdf]

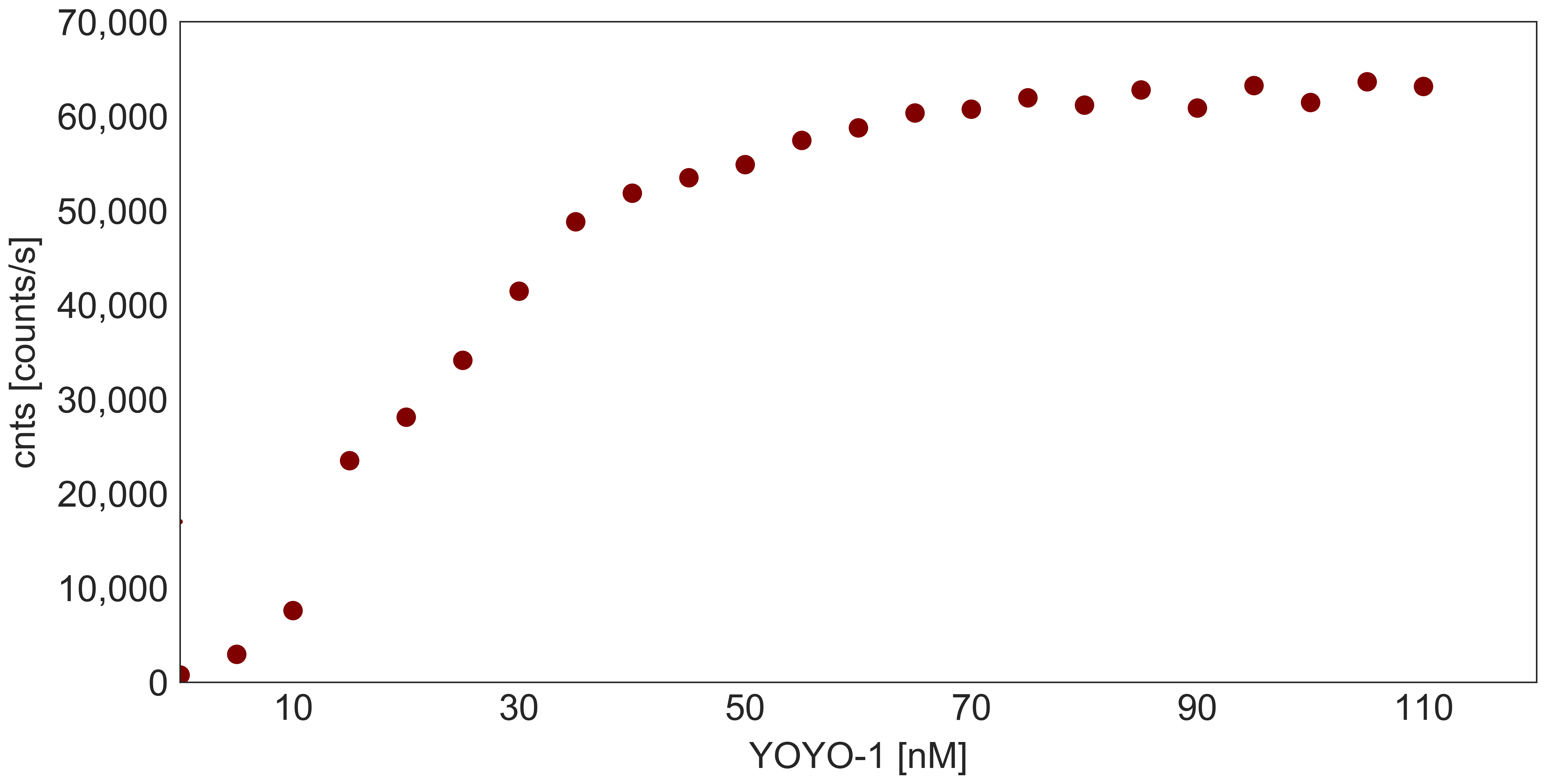

Supplement: Supplementary file 1 [file molecules-26-03748-s001.zip › molecules-1253971-supplementary-proofread/DNA_const_f.png]

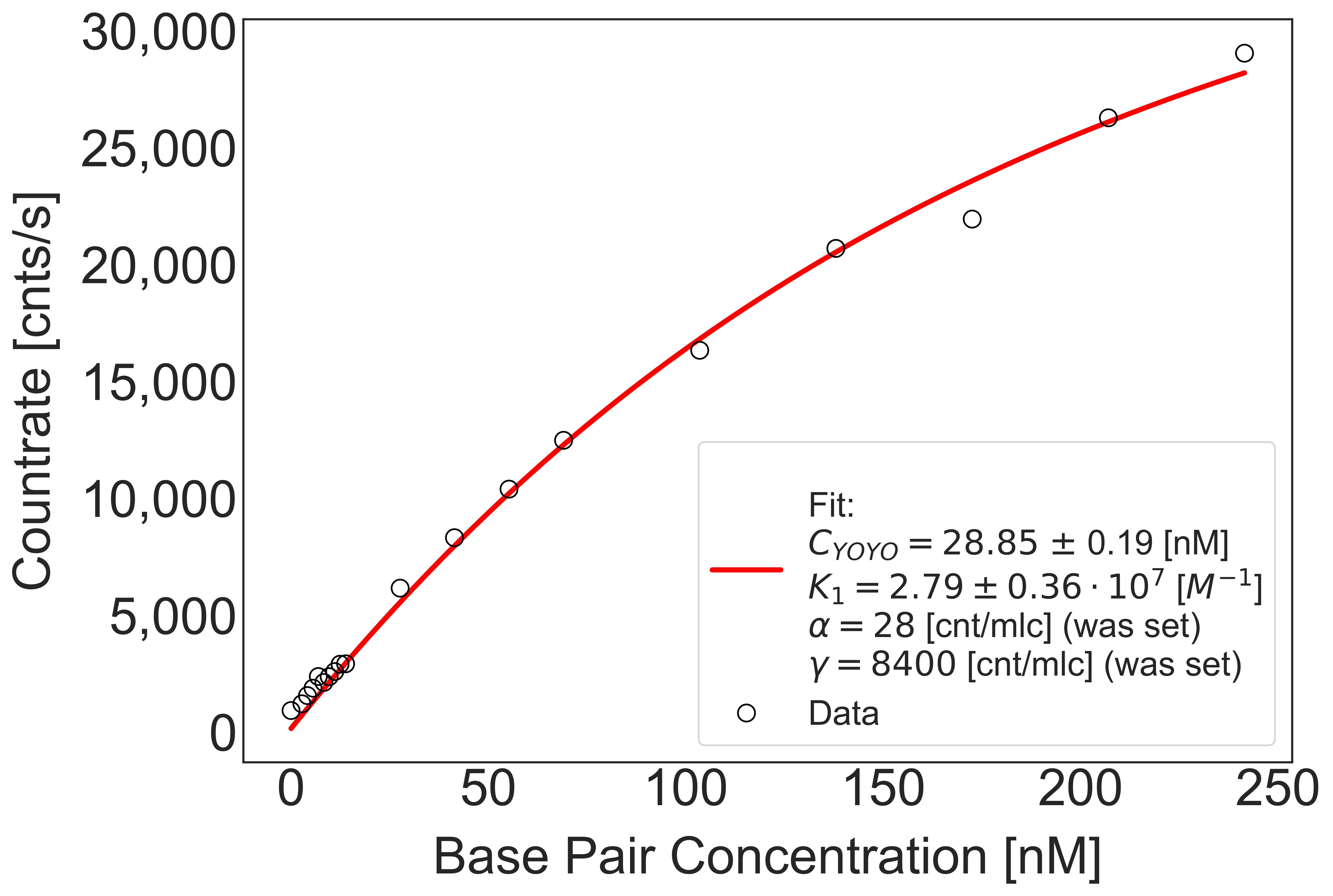

Supplement: Supplementary file 1 [file molecules-26-03748-s001.zip › molecules-1253971-supplementary-proofread/K1_f.png]

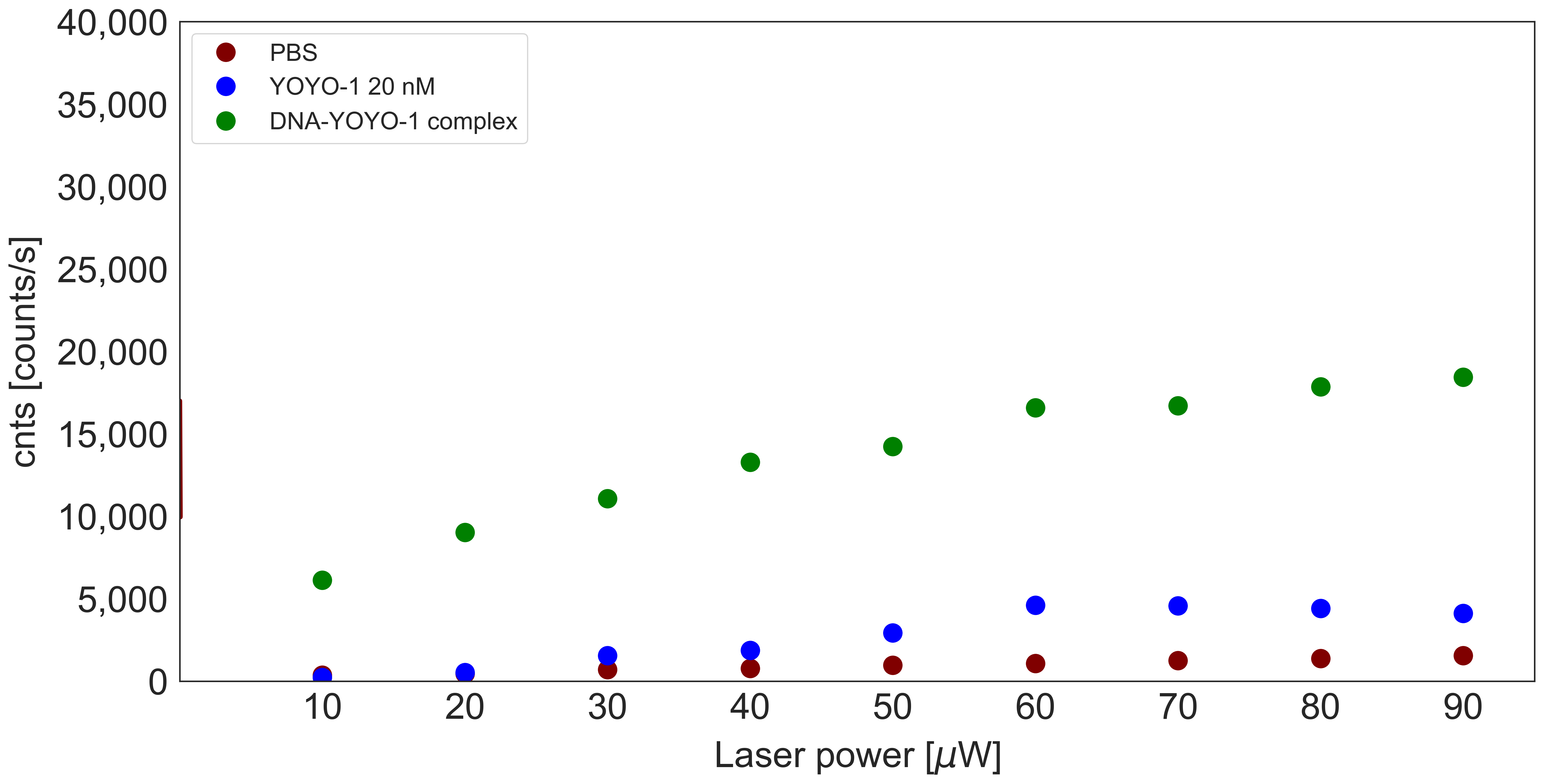

Supplement: Supplementary file 1 [file molecules-26-03748-s001.zip › molecules-1253971-supplementary-proofread/LP_f.png]

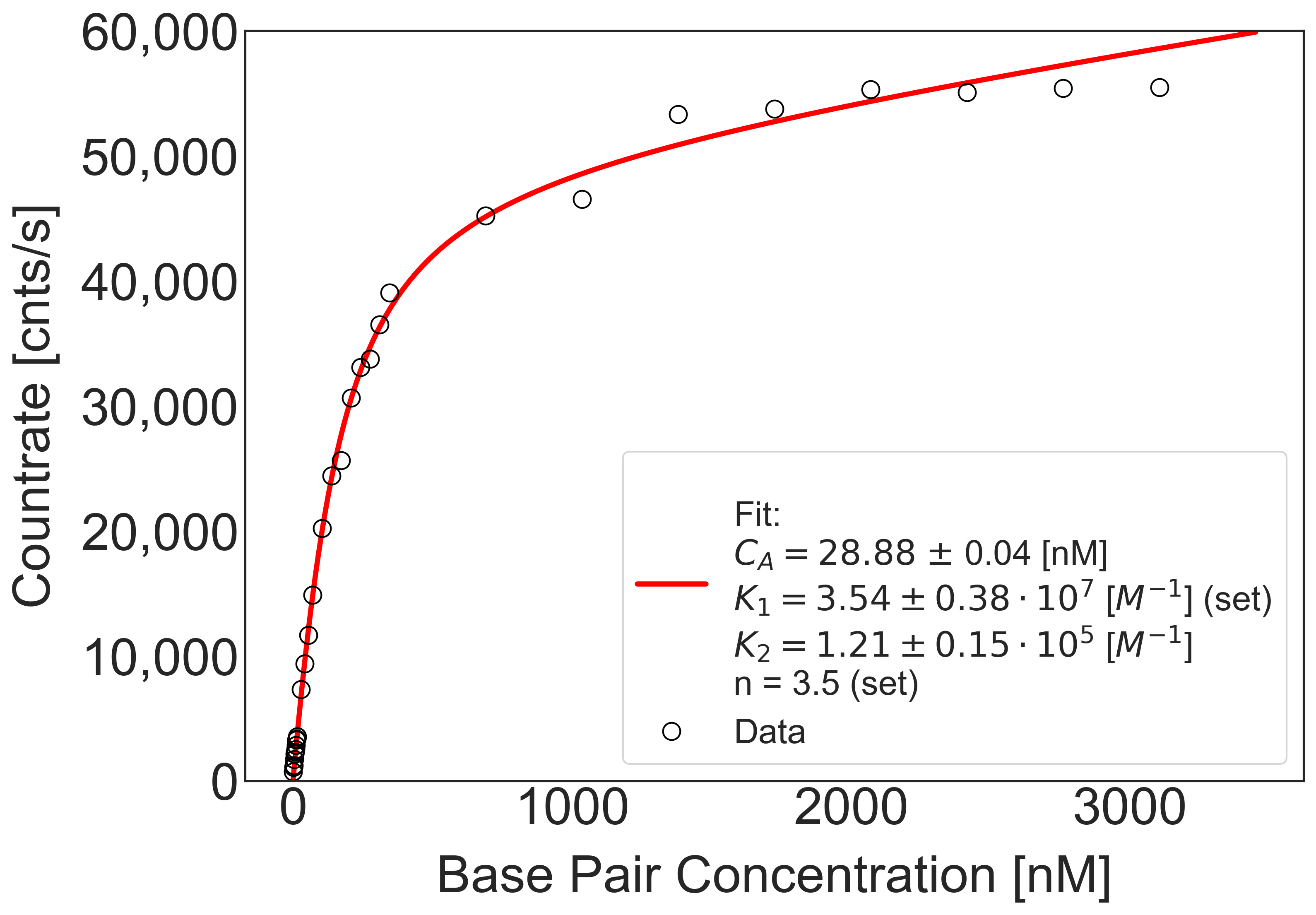

Supplement: Supplementary file 1 [file molecules-26-03748-s001.zip › molecules-1253971-supplementary-proofread/YOYO_second_step_8_06_n35.png]

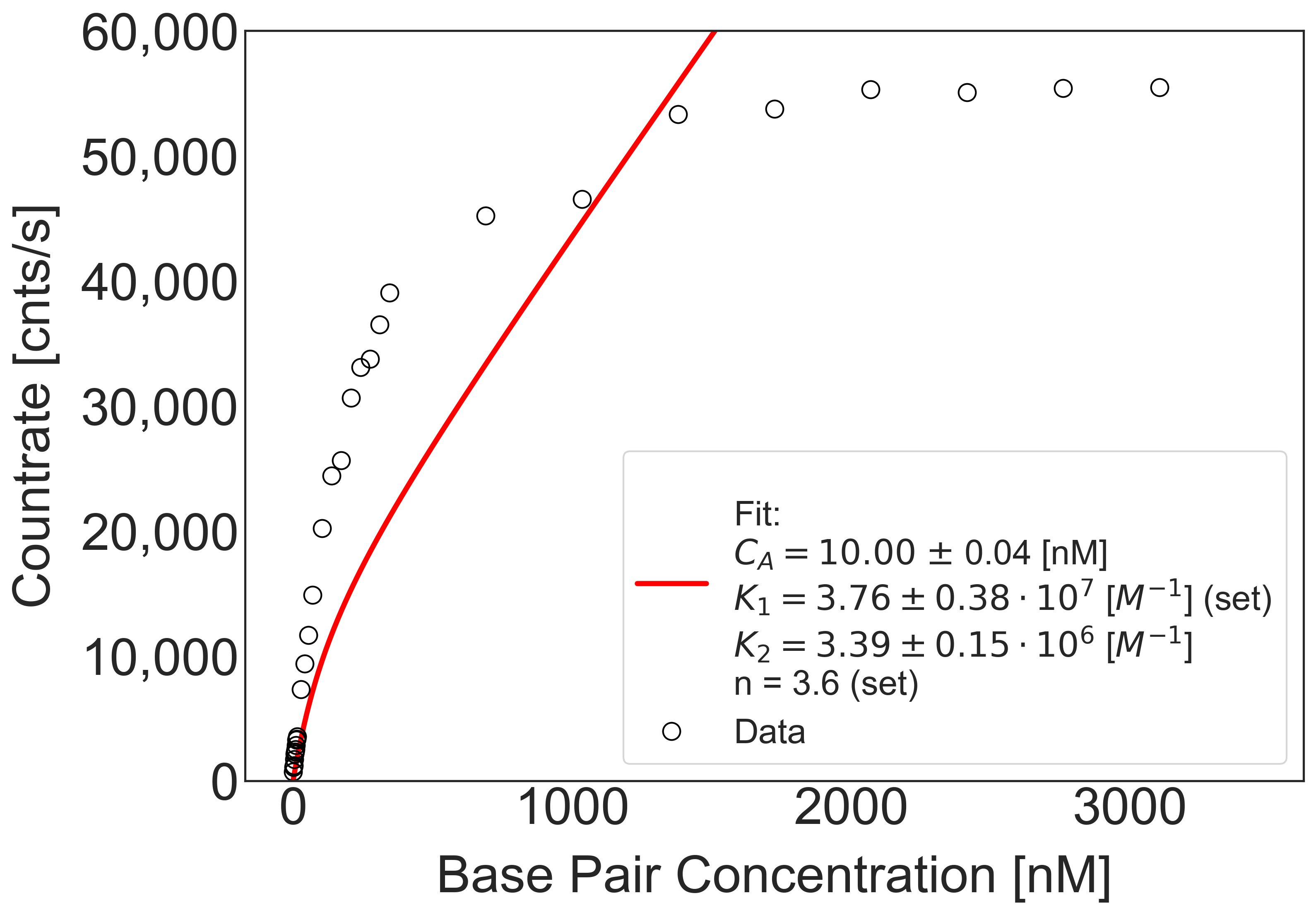

Supplement: Supplementary file 1 [file molecules-26-03748-s001.zip › molecules-1253971-supplementary-proofread/YOYO_second_step_8_06_n36f.png]
